# The Ball-Box Theorem for a Class of Corank 1 Non-differentiable Tangent Subbundles

**DOI:** 10.1007/s10883-018-9400-4

**Published:** 2018-05-04

**Authors:** Sina Türeli

**Affiliations:** Faculty of Mathematics, Imperial Collage London, London, UK

**Keywords:** Sub-Riemannian geometry, Dynamical systems, Non-differentiable bundles, Ball-box theorem, Primary 53Cxx, 53C17

## Abstract

We show that an analogue of the ball-box theorem holds true for a class of corank 1, non-differentiable tangent subbundles that satisfy a geometric condition. We also we give examples of such bundles and give an application to dynamical systems.

## Introduction

Sub-Riemannian geometry is a generalization of Riemannian geometry which is motivated by very physical and concrete problems [1, 2]. One drawback of current sub-Riemannian geometry literature is that it almost exclusively focuses on the study of “smooth systems”, which is sometimes too much to ask for a mathematical subject that has close connections with physical sciences. For instance, one place where non-differentiable objects appear in a physically motivated mathematical branch (and which is the main motivation of the authors) is the area of dynamical systems. More specifically in (partially and uniformly) hyperbolic dynamics, bundles that are only Hölder continuous are quite abundant and their sub-Riemannian properties (i.e. accessibility and integrability) play an important role in the description and classification of the dynamics. For example when one wants to study statistical properties, such as decay of correlations of Anosov flows, it becomes essential to give a quantitative relation between “non-integrability amount” and the amount one can travel in the horizontal direction via accessible curves. This appears in [18] as what is called a temporal function (the amount one travels in the transversal directions by following stable and unstable paths) and which is used to obtain one of the most critical inequalities in the paper the Dolgopyat inequality (see Section 6 and the Appendixes A and B in [18]). This relation is precisely the ball-box theorem in codimension 1. Since this tool is unavailable in the non-differentiable domain, results on decay of correlations for Anosov flows are restricted to the cases where the stable-unstable bundles are *C*^1^ contact bundles. Another place where sub-Riemannian geometry pops up in dynamical systems is in [7]. Here where they prove the integrability of bunched stable-center bundles (see Section 3.2 of this paper for definitions and Theorem 4.1 of [7]), the fact that one can travel along the transversal direction of a non-integrable bundle by using curves tangent to this bundle is the essential tool. Motivated by these applications, the aim of this paper is to give a little nudge to sub-Riemannian geometry in the direction of non-differentiable objects in an attempt to introduce sub-Riemannian geometry as a tool in dynamical systems.

Let Δ be a *C*^*r*^ ($r \geqslant 0$) tangent subbundle defined on a smooth manifold *M* and *g* a metric on Δ (the triple (*M*,Δ,*g*) is called a *C*^*r*^ sub-Riemannian manifold). We will always assume that Δ is corank 1 and *dim*(*M*) = *n* + 1 with $n \geqslant 2$. A piecewise *C*^1^ path *γ* is said to be *admissible* if it is a.e. tangent to Δ (i.e. $\dot {\gamma }(t) \in {\Delta }_{\gamma (t)}$ for *t* a.e.). We let $\mathcal {C}_{pq}$ denote the set of length parameterized (i.e. $g(\dot {\gamma }(t),\dot {\gamma }(t))= 1$ for *t* a.e.) admissible paths between *p* and *q*. If $\mathcal {C}_{pq} \neq \emptyset $ for all *p*, *q* ∈ *U* ⊂ *M*, then Δ is said to be *controllable* or *accessible* on *U*. For smooth bundles, the *Chow-Rashevskii theorem* says that if Δ is everywhere completely non-integrable (i.e. if the smallest Lie algebra *Lie*(Γ(Δ)) generated by smooth sections Γ(Δ) is the whole tangent space at every point), then it is accessible. In particular, if Γ(Δ)(*p*) + [Γ(Δ),Γ(Δ)](*p*) = *T*_*p*_*M*, then such a bundle is called step 2, completely non-integrable at *p*.^1^ We denote $d_{{\Delta }}(p,q) = \inf _{\gamma \in \mathcal {C}_{pq}}\ell (\gamma )$, where *ℓ*(⋅) denotes length with respect to the given metric *g* on Δ. *d*_Δ_ is called the *sub-Riemannian distance*. We let *B*_Δ_(*p*, *𝜖*) denote the ball of radius *𝜖* around *p* with respect to the distance *d*_Δ_. Given *p* ∈ *M* and a coordinate neighbourhood *U*, coordinates *z* = (*x*^1^,…,*x*^*n*^, *y*) are called *adapted* at *p* for Δ if, *p* = 0, ${\Delta }_{0} = \text {span} \left \langle \frac {\partial }{\partial x^{i}}|_{0} \right \rangle _{i = 1}^{n}$ and Δ_*q*_ is transverse to $\mathcal {Y}_{q} = \text {span} \left \langle \frac {\partial }{\partial y}|_{q} \right \rangle $ for every *q* ∈ *U*. Given such coordinates and *α* > 0, we define the coordinate weighted box as follows:

$$B_{\alpha}(0,\epsilon)=\{z=(x,y) \in U \quad | \quad |x^{i}| \leqslant \epsilon, \quad |y| \leqslant \epsilon^{\alpha}\}, $$

where |⋅| when applied to a vector denotes the Euclidean norm with respect to the given adapted coordinates and when applied to scalars is the usual absolute value. A specialization of the fundamental *ball-box theorem* [4, 20] says that if Δ is a smooth, step 2, completely non-integrable bundle at a point *p*, then, given any smooth adapted coordinate system defined on some small enough neighbourhood of *p*, there exists constants *K*_1_, *K*_2_, *𝜖*_0_ > 0 such that for all *𝜖* < *𝜖*_0_,

1.1 $$ B_{2}(0,K_{1}\epsilon) \subset B_{{\Delta}}(0,\epsilon) \subset B_{2}(0, K_{2}\epsilon).  $$

Note that here the lower inclusion *B*_2_(0,*K*_1_*𝜖*) ⊂ *B*_Δ_(0,*𝜖*) implies accessibility around *p*. This special case is also known to hold true for *C*^1^ bundles (see [12]). From now on, when we say the ball-box theorem, we mean the version for step 2 bundles as described above(in particular our bundles will be codimension 1).

For proving continuous versions of this theorem, smooth approximation techniques are generally not sufficient. This is due to two reasons: First, the constants *K*_1_, *K*_2_, *𝜖*_0_ and the neighbourhood appearing in the theorem are usually very implicit. Second, in rare cases when not, they turn out to depend on the *C*^1^ or Lipschitz norms of vector-fields that are tangent to the subbundle. *C*^1^ norms are useless when it comes to smooth approximation approach for non-differentiable objects. As far as we are aware, one of the only proofs where the constant *K*_2_ is related to something more flexible than the *C*^1^ norm is given in [12]. There the bundle Δ is assumed to be *C*^1^ and for Δ = *ker*(*η*), *K*_2_ is related to *C*^0^ norm of *dη* via Stokes’ theorem. In our opinion, Stokes’ theorem and *C*^0^ norm of *dη* is much well behaved than *C*^1^ norms (see [14] or [19]). This is what allows the author of [23] to apply this method to Hölder continuous bundles and obtain the upper inclusion “*B*_Δ_(0,*𝜖*) ⊂ *B*_*α*_(0,*K*_2_*𝜖*)” with *α* = 1 + *𝜃*. However, they can not prove the lower inclusion; therefore, one can not get any description of the volume of the sub-Riemannian ball nor a criterion for accessibility. In particular, one can not readily produce any examples of bundles that satisfy this theorem. One of the missing ingredients for the lower inclusion is that there is no geometric proof for the differentiable cases that uses something better than the *C*^1^ norm.

In this paper, we prove the ball-box theorem for a class of continuous bundles (which include even non-Hölder examples as well as all *C*^1^ bundles). Motivated by the discussion above, our proof is very geometric and uses Stokes’ theorem as the main tool rather than classical ODE analysis. The techniques that we use are inspired from a proof of the Frobenius theorem given in [3] and a proof of the upper inclusion of ball-box theorem in [12]. We pay special attention to understanding how the constants *K*_1_, *K*_2_ appearing in the theorem are related to geometric properties of the bundle such as the *C*^0^ norm of *dη*. This is because we believe that the geometric method of proof presented here will pave the way to proving this theorem for a more general class of non-differentiable bundles than presented here. After the proof of the main theorems, we construct examples of bundles that satisfy these geometric properties and yet are not differentiable (nor Hölder). Finally, we give an application to dynamical systems.

In the passing, we note that there are few other works in sub-Riemannian literature that take into account lower regularity bundles. In [21], they prove the full ball-box theorem under certain Lipschitz continuity assumptions for commutators of the vector fields involved and in particular for Δ. In [17], they work with a slightly different language than here or [21] but it can be seen as a generalization of the result in [12] to arbitrary steps and in particular assumes Δ is *C*^1^. These results do not apply to the class of examples that we can study with the main theorem of this paper. However, the main theorem in this paper does not cover these theorems as well since we can only consider step 2 cases while these results consider general step cases.

### Statement of the Theorems

We will carry out the proof in coordinate neighbourhoods. The domain of the coordinate will be possibly a smaller Euclidean box where we work and all the supremums and infumums of the functions defined on this coordinate neighbourhood will be over this domain. We denote the domain of any chosen coordinate system by $U \subset \mathbb {R}^{n + 1}$. We identify the tangent spaces with $\mathbb {R}^{n + 1}$. We let ${{\Omega }^{n}_{r}}(U)$ denote the space of *C*^*r*^ differential *n*-forms over *U*. We denote by $\mathcal {A}^{n}_{r}({\Delta })(U) \subset {{\Omega }^{n}_{r}}(U)$ the space of *C*^*r*^ differential *n*-forms over *U* that annihilate Δ. We use the induced norm on these spaces coming from the Euclidean norm. We use |⋅|_*∞*_ and |⋅|_inf_ denote the supremum and infimum of the norms of an object over the domain *U* we are working with. Given a *C*^*r*^ ($r \geqslant 0$) differential *k* − *formα*, we denote:

$$|\alpha|_{{\Delta}}|_{\infty} = \underset{q \in U}{\sup} \underset{v_{i} \in {\Delta}_{q}, \ |v_{1} \wedge {\dots} \wedge v_{k}|= 1}{\sup} |\alpha_{q}(v_{1} \wedge {\dots} \wedge v_{k})|, $$

$$|\alpha|_{{\Delta}}|_{\inf} = \underset{q \in U}{\inf} \underset{v_{i} \in {\Delta}_{q}, \ |v_{1} \wedge {\dots} \wedge v_{k}|= 1}{\sup} |\alpha_{q}(v_{1} \wedge {\dots} \wedge v_{k})|. $$

The first expression is the supremum over *U* of the norms of *α*_*q*_ seen as linear functionals on $\bigwedge ^{k} ({\Delta }_{q})$ while the second is the infimum over *U* of the same expression. In proving the ball-box type theorems, we can for simplicity replace *g* locally by the Euclidean metric (since their norms are equivalent). In particular from now on, *d*_Δ_ is calculated with respect to |⋅|. Now, we define the main property that we need:

#### **Definition 1.1**

A continuous differential 1-form *η* is said to have a continuous exterior derivative^2^ if there exists a continuous differential (2)-form *β* such that for every differentiable 1-cycle *Y* and differentiable 2-chain *H* bounded by it,^3^ one has that

$${\int}_{Y} \eta = {\int}_{H} \beta. $$

If such a *β* exists, we suggestively denote it as *dη*. A rank 1 subbundle $\mathcal {E} \subset {{\Omega }_{0}^{n}}(M)$ is said to be equipped with continuous exterior derivative at *p* ∈ *M*, if there exists a neighbourhood *V* of *p* on which *η* is a local basis of sections of $\mathcal {E}$ on *V* and *dη* is its continuous exterior derivative. We will refer to above property as Stokes’ property and also denote this triple by {*V*, *η*, *dη*}. For $\mathcal {E}={\mathcal {A}}_{0}^{1}({\Delta })(M)$, we will sometimes say shortly that Δ is equipped with a continuous exterior derivative.

#### **Definition 1.2**

Let Δ be a corank 1, continuous, tangent subbundle such that ${\mathcal {A}}_{0}^{1}({\Delta })(M)$ is equipped with a continuous exterior derivative {*V*, *η*, *dη*} at *p*_0_. We say that Δ is non-integrable at *p*_0_ if

$$(\eta \wedge d\eta)_{p_{0}} \neq 0. $$

We will now give an analogue of the ball-box theorem which also has the usual *Chow-Rashevskii theorem* (for the class of bundle, we consider in this paper) as its corollary. In the following, we assume that Δ has modulus of continuity *ω* (where *ω* : [0,*∞*) → [0,*∞*) is an increasing function with *ω*(0) = 0). This means that given any neighbourhood *U* ⊂ *M* and any coordinate system on *U*, Δ can be locally spanned by vector fields $\{Y_{i}\}_{i = 1}^{n}$ such that in the Euclidean norm of the coordinates:

$$|Y_{i}(q)-Y_{j}(p)| \leqslant C\omega(|p-q|). $$

for some constant *C* > 0. For simplicity of notation, we denote for any $r \in \mathbb {R}^{+}$, *ω*_*r*_ = *ω*(*r*). In an adapted coordinate system with a domain *U*, we can also define a basis of sections Δ of the form

**Fig. 1:**
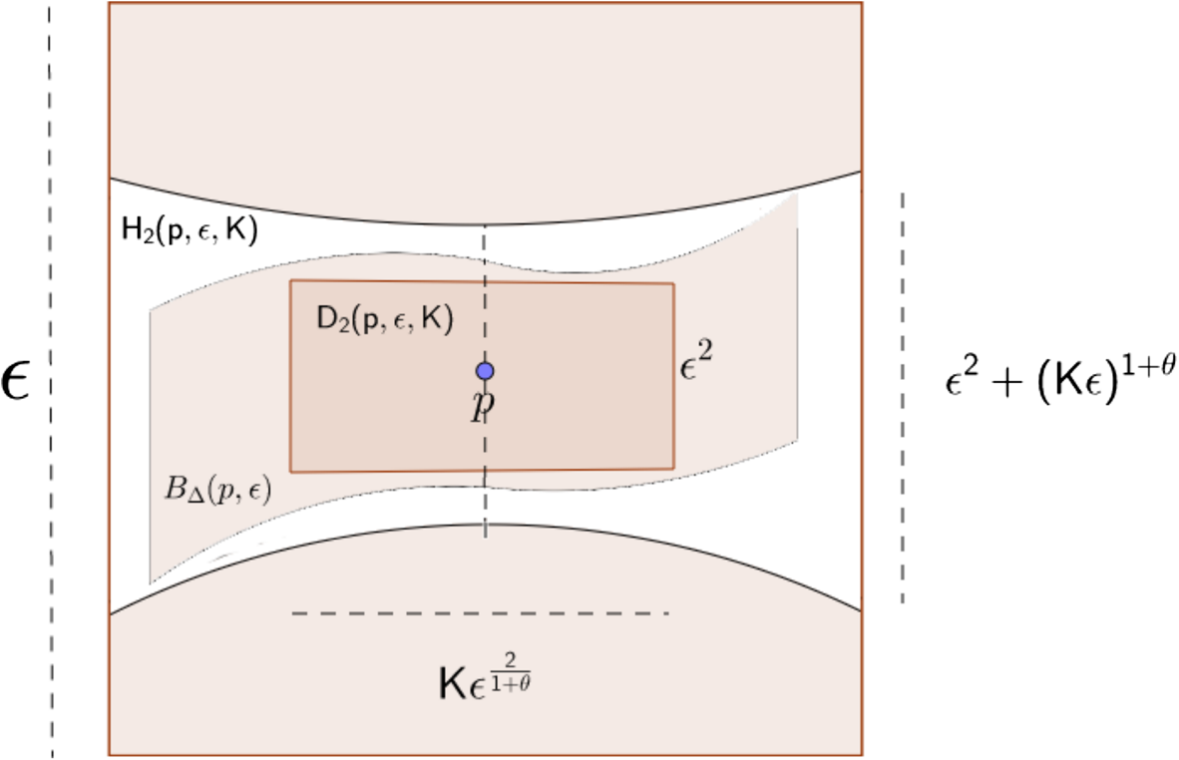
Pictorial representation of Theorem 1 with *ω*(*t*) = *t*^*𝜃*^

1.2 $$ X_{i} = \frac{\partial}{\partial x^{i}} + a_{i}(x,y)\frac{\partial}{\partial y},  $$

where *a*_*i*_(*x*, *y*) are continuous functions. The functions *a*_*i*_ have modulus of continuity $\tilde {C}\omega $ on *U* with respect to |⋅|, with some multiplicative constant $\tilde {C}>0$ possibly depending on the chosen coordinates. The assumption of non-integrability at *p*_0_ would then mean that there exists *i*, *j* ∈{1,…,*n*}, *i*≠*j* and a domain *U* such that |*dη*(*X*_*i*_, *X*_*j*_)|_inf_ > 0. Therefore, we also have |*dη*|_Δ_|_inf_ > 0. Also given some adapted coordinates (*x*^1^,…,*x*^*n*^, *y*) for the bundle Δ, we define the following:

$${D}_{2}^{\omega}(0,K_{1},\epsilon)=\left\{z=(x,y) \in U \quad | \quad |x| + \sqrt{\frac{1}{K_{1}}(|x|\tilde{C}\omega_{2|x|} +|y|)} \leqslant \epsilon \right\}, $$

$${H}_{2}^{\omega}(0,K_{2},\epsilon)=\{z=(x,y) \in U \quad | \quad |x| \leqslant \epsilon, \quad |y| \leqslant K_{2}\epsilon^{2}+ |x|\tilde{C}\omega_{2|x|}\}, $$

$$\mathcal{B}_{2}(0,K_{3},\epsilon)=\{z=(x,y) \in U \quad | \quad |x| \leqslant \epsilon, \quad |y| \leqslant K_{3}\epsilon^{2}\}, $$

where $|x| = {\sum }_{i = 1}^{n} |x_{i}|$ (see Fig. 1)). Finally when needed, we denote the generic constants that depends only on the dimension of the manifold and on $\tilde {C}$ by capital letters such as *C*(*n*), *D*(*n*) and *E*(*n*). The main theorem is as follows:

#### **Theorem 1**

*Let* Δ *be a corank 1, continuous tangent subbundle with modulus of continuity* *ω*, *equipped with a continuous exterior derivative* {*V*, *η*, *dη*} *at* *p*_0_ *and that* Δ *is non-integrable at* *p*_0_. *Then, for any smooth adapted coordinate system around* *p*_0_, *there exists a domain* *U and constants* *𝜖*_0_, *K*_1_, *K*_2_ > 0 *such that, for all* *𝜖* < *𝜖*_0_*:*

1.3 $$ {D}_{2}^{\omega}\left( 0, K_{1},\frac{1}{4}\epsilon\right) \subset B_{{\Delta}}(0,\epsilon) \subset {H}_{2}^{\omega}(0,K_{2},\epsilon),  $$

*where* *K*_1_, *K*_2_ > 0 *are constants given by*

$$K_{1} = \frac{1}{C(n)}\frac{|d\eta|_{{\Delta}}|_{\inf}}{|\eta(\partial_{y})|_{\infty}}, \quad \quad K_{2} = C(n) \frac{|d\eta|_{{\Delta}}|_{\infty}}{|\eta(\partial_{y})|_{\inf}}, $$

*for some* *C*(*n*) > 0. *Moreover, there exists a* *C*^1^ *adapted coordinate system such that*,

$$\mathcal{B}_{2}\left( 0,K_{1}, \frac{\epsilon}{4}\right) \subset B_{{\Delta}}(0,\epsilon) \subset \mathcal{B}_{2}(0,K_{2},\epsilon). $$

#### **Corollary 1.3** (Chow-Rashevskii Theorem)

*Let* Δ *be a corank 1, continuous tangent subbundle* *equipped with a continuous exterior derivative*{*V*, *η*, *dη*} *at* *p*_0_. *If* Δ *is non-integrable at* *p*_0_, *then it is accessible in some neighbourhood of* *p*_0_.

#### Remark 1.4

One might question the reason behind in trying to obtain an estimate in smooth coordinates when the bundle itself is not differentiable. In questions which are in the domain of interest of the authors where sub-Riemannian geometry can be potentially applied, smooth and non-differentiable objects coexist. For instance, it might be that a smooth Anosov flow f might exist together with only a Hölder continuous bundle. Therefore, even though the bundle might be non-differentiable, estimates obtained in smooth coordinates might be important. Also note that unlike the smooth ball-box theorem, this theorem does not make a statement about all *C*^1^ adapted coordinates. It can however do so for all smooth coordinates with some loss on the power of the ball-box estimates. Finally, although the upper inclusion looks similar to the one in [12], one essential difference is that |*dη*|_*∞*_ has been replaced by |*dη*|_Δ_|_*∞*_. We believe that the technique that gives this result will allow us to extend the theorem to the cases where *dη* might not exits but *dη*|_Δ_ exists in some appropriate sense.

#### Remark 1.5

This theorem is a generalization of the smooth ball-box theorem on corank 1, step 2 bundle case. Indeed, if Δ is smooth, then *ω*_*t*_ ≤ *Kt* and plugging this into equations defining ${D}_{2}^{\omega }$ and ${H}_{2}^{\omega }$, one obtains boxes.

#### Remark 1.6

Later on, we will see that the size of U and *𝜖*_0_ depends mainly on *ω*, |*dη*|_*∞*_ and |*dη*|_Δ_|_*inf*_. This observation will be useful for anyone who wants to apply or generalize these results to a broader class of examples via approximation techniques and this will be the content of upcoming papers. In the proof of this theorem, we do not use approximation techniques but rather work with the continuous bundle directly. In principle, one could apply the methods in this paper to a sequence of differentiable one forms *η*^*k*^ such that they only converge to *η* in *C*^0^ norm (so their derivatives blow up) and also that *dη*^*k*^ converges. The techniques in this paper will allow one to quantify the constants and the sizes of the neighbourhoods in terms of *ω*, |*dη*|_*∞*_ and |*dη*|_Δ_|_*inf*_. This will however come with the additional difficulty of bookkeeping another index for the sequence and deal with more error terms that comes from the distance of the approximating basis to the limiting ones (which will still have to be defined). Therefore, we choose to give the proof without approximation and by directly working with the bundle itself.

We also note that the classical proofs for ball-box theorem for differentiable bundles are not sufficient for using in an approximation method. This is mainly because even when one finds a proof where the dependence of constants are laid out explicitly, they depend on the *C*^1^ or Lipschitz norms of the objects that are involved (see for instance [21]). In fact, it is one of the main motivations of this paper to relate the constants appearing in ball-box theorem to |*dη*^*k*^|, something much better than the *C*^1^ norm. This paper together with a previous paper [19] by the author and works of Hartman shows that to study important geometric and analytic properties of vector fields and bundles it is sufficient to consider “exterior derivative norms” rather than *C*^1^ norms. To support this further we give examples of bundles which satisfy this regularity condition but are not differentiable (see Section 3).

### Organization of the Paper

In this subsection, we describe the layout of the paper and the main ideas of the proofs.

Section 2 contains the proof of Theorem 1. The proof of this theorem has two main ingredients. These are the fundamental tools that we use repeatedly and are therefore explained in separate Sections 2.1 and 2.2. First ingredient is Proposition 2.1, which states that the adapted basis is uniquely integrable. This is only due to existence of the continuous exterior derivative and can readily be derived from results of Hartman in [14]. The second ingredient is Proposition 2.4 which relates the amount admissible curves travel in the *∂*_*y*_ direction to a certain surface integral of *dη*. This again only assumes existence of the continuous exterior derivative and Proposition 2.1. This proposition can be seen as a generalization of certain artful constructions from [3] (see Section 36 of Chapter 7 and Appendix 4).

Then, in Section 2.3, we prove Theorem 1 using Propositions 2.1 and 2.4. The main idea is to first construct certain accessible *n*-dimensional manifolds $\mathcal {W}_{\epsilon }$ (see Lemma 2.7) and study how the sub-Riemannian balls are spread around them (see Lemma 2.8).

To summarize the main ideas of the proof of Theorem 1, we can say

- The existence of *dη* and the condition that *η* ∧ *dη* ≠ 0 gives volume to the sub-Riemannian ball.
- The loss of regularity in the bundle may cause the sub-Riemannian ball to bend which results in the outer sub-Riemannian ball being distorted and the inner one getting smaller.

In Section 3, we give some basic properties of continuous exterior derivative and give some examples of non-differentiable and even non Hölder bundles and satisfy the conditions of Theorem 1. We also give an application to dynamical systems.

## The Proof

### Proposition 2.1

We call a continuous vector-field uniquely integrable if it is integrable and if *γ*_1_ and *γ*_2_ are two integral curves such that *p* ∈ *γ*_1_ ∩ *γ*_2_, then *p* is contained in a relatively open (in both integral curves) subset of *γ*_1_ ∩ *γ*_2_. In this case, there exists a unique maximal integral curve of *X* (not to be confused with maximal and minimal solutions of non-uniquely integrable vector-fields), starting at *q* and defined on the interval [−*𝜖*_*q*_, *𝜖*_*q*_]. We denote this integral curve by *t* → *e*^*tX*^(*q*).

We say that an integrable vector field *X* defined on *U*_1_ has *C*^*r*^ family of solutions if there exists some *U*_2_ ⊂ *U*_1_ and an *𝜖*_0_ such that for all *p* ∈ *U*_2_, there exists an integral curve passing through *p* with *𝜖*_*p*_ ≥ *𝜖*_0_ so that this choice of solutions seen as maps from [−*𝜖*_0_, *𝜖*_0_] × *U*_2_ → *U*_1_ are *C*^*r*^.

Next proposition is almost a direct corollary of the Theorem 6.1 [14] (Chapter 5, Section 6). However, the terminology used there is quite different from here and the proof uses classical ODE analysis and approximation techniques. While it is possible to give a proof based on Stokes’ property, it is rather lengthy so we avoid that. Instead, following our proposition, we will state the theorem in [14] and show how it translates to ours.

#### **Proposition 2.1**

*Let* Δ *be a corank 1, continuous tangent subbundle such that, at* *p*_0_, ${\mathcal {A}}_{0}^{1}({\Delta })(M)$ *is* *equipped with a continuous exterior derivative* {*V*, *η*, *dη*}. *Then, for any adapted coordinate* {*x*^1^,…,*x*^*n*^, *y*} *around* *p*_0_ *with an adapted basis* $\{X_{i}\}_{i = 1}^{n}$, *there exists some domain* *U on which* *X*_*i*_ *are uniquely integrable and have* *C*^1^ *family of solutions.*

The Theorem 6.1 in [14] is as follows:

#### **Theorem 2**

*Let* $f(t,y,z): U\subset \mathbb {R}^{m+n + 1} \rightarrow \mathbb {R}^{n}$ *(with* $y \in \mathbb {R}^{n}$, $z \in \mathbb {R}^{m}$*)* *be continuous. Then, the ODE* $\frac {dy}{dt}=f(t,y,z)$ *has* *unique and* *C*^1^ *solutions* *y*(*t*) = *η*(*t*, *t*_0_, *y*_0_, *z*),*y*(*t*_0_) = *y*_0_ *for all* (*t*_0_, *y*_0_, *z*) ∈ *U*, *if for any* *p* ∈ *U*, *there exists a neighbourhood* *U*_*p*_, *a non-singular* *n* × *n* *matrix* *A*(*t*, *y*, *z*) *and a continuous* *n* × *m* *matrix* *C*(*t*, *y*, *z*) *such that the* 1 −*forms* $\eta ^{i} = {\sum }_{i = 1}^{n} A^{ij} (dy^{i} - f^{i}dt) + {\sum }_{j = 1}^{m} C^{ij}dz^{j}$ *have* *continuous exterior derivative.*

#### Remark 2.2

This theorem considers the general case of an external parameter-dependent ODE where the external parameter is (*z*^1^,…,*z*^*m*^). This generality is not needed for our purposes; therefore, we assume that *m* = 0 (see Equations 1.1 and 1.2 in Chapter 5 of [14]). For this reason, we will also take *C* = 0 and drop any dependency on z.

#### *Proof of Proposition 2.1, assuming Theorem 2*

We start by translating the terminology of Theorem 2 to something more closer to ours. Note that the unique integrability of the non-autonomous ODE $\dot {y}=f(t,y)$ with *C*^1^ solutions above is equivalent to unique integrability of the extended vector field $X=\frac {\partial }{\partial t} + {\sum }_{i = 1}^{n} f^{i}(t,y)\frac {\partial }{\partial y^{i}}$ with *C*^1^ family of integral curves which would be given by *t* → (*t*, *η*(*t*, *t*_0_, *y*_0_)) ⊂ *U* for t small enough. Let $\mathbb {X}$ be the bundle spanned by this vector field in the (*t*, *y*) space. Then, this bundle is the intersection of the kernel of the 1-forms *η*^*i*^ = *dy*^*i*^ − *f*^*i*^*dt*, i.e $\mathbb {X}=\cap _{i = 1}^{n} ker(\eta ^{i})$. Therefore, *η*^*i*^ is a basis of sections for $\mathcal {A}_{0}^{1}(\mathbb {X})$. In particular, then, the condition of Theorem 2 about the existence of a non-singular *A*(*t*, *y*) simply asks that there must exist a basis of sections for $\mathcal {A}_{0}^{1}(\mathbb {X})$ (which is given by $\alpha ^{i}={\sum }_{i = 1}^{n} A^{ij} (dy^{i} - f^{i}dt)$) which has continuous exterior derivative. Following the Definition 1.1, this means that if the line bundle $\mathbb {X}$ is equipped with a continuous exterior derivative, then it is uniquely integrable with *C*^1^ solutions. To prove Proposition 2.1, we will show that the conditions given there imply that each vector field *X*_*i*_ is equipped with a continuous exterior derivative. We have that

$$X_{i} = \frac{\partial}{\partial x^{i}}+ a_{i}(x,y)\frac{\partial}{\partial y}. $$

Let $\mathbb {X}_{i}$ be the bundle spanned by this non-vanishing vector-field. We set *α*^*j*^ = *dx*^*j*^ for *j*≠*i* and *α*^*i*^ = *η* which is the 1-form given in the proposition. Note that $\{\alpha ^{j}\}_{j = 1}^{n}$ forms a linearly independent basis for $\mathcal {A}_{0}^{1}(\mathbb {X}_{i})$. Indeed, $dx^{j}(X_{i})={\delta ^{j}_{i}}$ and *η*(*X*_*i*_) = 0 since *η* annihilates Δ which contains *X*_*i*_. Moreover, by our choice of adapted coordinates, *η*≠*dx*^*i*^ on some neighbourhood around *p*_0_. Now by assumption, *η* has continuous exterior derivative and *dx*^*j*^ has continuous exterior derivative 0, so $\mathcal {A}_{0}^{1}(\mathbb {X}_{i})$ has a basis whose elements have continuous exterior derivative. This proves that each *X*_*i*_ is uniquely integrable. □

With this proposition, once a suitable neighbourhood *U* is given, we can find $\tilde {U} \subset U$ and *𝜖*_0_ be such that for all *k* = 1,...*n*, $q \in \tilde {U}$, *t* ≤ *𝜖*_0_, $e^{tX_{k}}(q)$ is uniquely defined and *C*^1^. We will impose some more conditions which will fix *U* once and for all for the rest of the paper. The conditions are as follows:

- For all, *k* = {1,…,*n*}
  2.1 $$ \frac{1}{2} \leqslant |X_{k}|_{\inf} \leqslant |X_{k}|_{\infty} \leqslant 2 \quad \text{and} \quad |X_{1} \wedge {\ldots} \wedge X_{n} \wedge \partial_{y}|_{\inf} \geqslant \frac{1}{2}.  $$
- For some, *i*, *j* ∈{1,…,*n*}
  2.2 $$ d\eta_{p}(X_{i}(p),X_{j}(p))\neq 0,  $$
  for all, *p* ∈ *U*.
- For some, *i*, *j* ∈{1,…,*n*}
  2.3 $$ |d\eta(X_{i},X_{j})|_{\inf} \geqslant B(n)|d\eta|_{{\Delta}}|_{\inf}  $$
  for all *p* ∈ *U* and for some constant *B*(*n*) > 0.

These are possible since *X*_*k*_(0) = *∂*_*k*_ and *η* is non-involutive at 0.

We will also do some modifications on *𝜖*_0_ starting with choosing it small enough so that $4n^{2}K_{2}{\epsilon ^{2}_{0}}<\epsilon _{0}$, where *K*_2_ is as in Theorem 1 and $\tilde {C}\omega _{2n\epsilon _{0}} \leqslant 1$. Then, we assume for all $q\in \tilde {U}$,

2.4 $$ B(q,20n\epsilon_{0})\subset U.  $$

One consequence of this and the fact that |*X*_*k*_|_*∞*_≤ 2 is that uniqueness and differentiablity of solutions is extended to *n* + 4-tuple compositions. That is for all $q \in \tilde {U}$, $|t_{i_{k}}| \leqslant \epsilon _{0}$, where *i*_*k*_ ∈{1,…,*n*} and *k* = 1,…,*n* + 4, one has that the following is well defined:

2.5 $$ e^{t_{i_{k}}X_{i_{k}}}\circ {\dots} \circ e^{t_{i_{1}}X_{i_{1}}}(q) \in U.  $$

and this composition of flows is a *C*^1^ map with respect to *q* and $t_{i_{k}}\neq 0$. Another consequence of the above conditions is that ${H}_{2}^{\omega }(0,K_{2},2n\epsilon ) \subset U$ for all *𝜖* ≤ *𝜖*_0_ where *K*_2_ is as in Theorem 1.

#### Remark 2.3

In the remaining part of the proof, there will be places where for some constants *E*(*n*),*G*(*n*) > 0 we fill shrink *𝜖*_0_ so that $E(n) \omega _{G(n) \epsilon _{0}} |d\eta |_{{\Delta }}|_{\infty } \leqslant |d\eta |_{{\Delta }}|_{\inf }$. We do not include these constants here explicitly since their exact form are quite complicated. Note that all the conditions above remain valid when we decrease the size of *𝜖*_0_ later on.

### Proposition 2.4

From now on, we will always be working with admissible curves *γ* : [0,*𝜖*] → *U* with *𝜖* ≤ *𝜖*_0_ that are either unit parameterized or satisfy $\dot {\gamma }(t)=X_{i}(\gamma (t))$ for all *t*. Therefore, the reader should keep in mind that for such curves $\frac {\epsilon }{2} \leqslant \ell (\gamma ) \leqslant 2 \epsilon $. We let Π_*x*_ be the projection to *x* coordinates.

#### **Proposition 2.4**

*Let* *γ*_*ℓ*_ : [0,*ε*_*ℓ*_] → *U* *for* *ℓ* = 1, 2 *be two* Δ −*admissible* *curves such that* *γ*_1_(0) = *γ*_2_(0) = *q*, Π_*x*_*γ*_1_(*ε*_1_) = Π_*x*_*γ*_2_(*ε*_2_), $\frac {1}{2} \leqslant |\dot {\gamma }_{\ell }|_{\inf } \leq |\dot {\gamma }_{\ell }|_{\infty } \leqslant 2$. *Let* *ε* = max{*ε*_1_, *ε*_2_} *and* *β* *be the segment in the* *∂*_*y*_ *direction that connects* *γ*_1_(*ε*_1_) *to* *γ*_2_(*ε*_2_). *Let* *α*_*ℓ*_ *be the 1-chains which are projections of* *γ*_*ℓ*_ *along* *∂*_*y*_ *to* Δ_*q*_ *and assume that* *α*_*ℓ*_ ⊂ *U*. *Then, for any 2-chain* *P* ⊂Δ_*q*_ ∩ *U* *whose boundary is* ${\alpha }_{1}^{-1} \circ \alpha _{2}$, *we have that*

2.6 $$ \frac{1}{|\eta(\partial_{y})|_{\infty}}\left( \left|{\int}_{P} d\eta \right| - |c|\right) \leqslant |\gamma_{1}(\varepsilon_{1}) - \gamma_{2}(\varepsilon_{2})| \leqslant \frac{1}{|\eta(\partial_{y})|_{\inf}}\left( \left|{\int}_{P} d\eta\right| + |c|\right),  $$

*and*

2.7 $$ sign\left( {\int}_{\beta}\eta\right) = sign\left( -{\int}_{P} d\eta + c\right), \quad \text{where} \quad |c| \leqslant C(n) \omega_{2\varepsilon}\varepsilon^{2} |d\eta|_{\infty}.  $$

#### Proof

Denote *γ*_1_(*ε*_1_) = *q*_1_, *γ*_2_(*ε*_2_) = *q*_2_. Assume w.l.o.g that $q_{1} \geqslant q_{2}$ with respect to the order given by the positive orientation of *∂*_*y*_ direction. Since *γ*_*ℓ*_ are admissible curves, we have that

$$\dot{\gamma}_{\ell}(t)= \sum\limits_{k = 1}^{n} {u}_{\ell}^{k}(t)X_{k}(\gamma_{\ell}(t)), $$

*t* a.e. for some piecewise *C*^0^ functions ${u}_{\ell }^{k}(t)$. Define the following non-autonomous vector fields

$$Z_{\ell}(t,p) = \sum\limits_{k = 1}^{n} {u}_{\ell}^{k}(t)X_{k}(q). $$

One has that *α*_*ℓ*_ are integral curves of *Z*_*i*_ starting at *t* = 0 and *q* which are inside Δ_*q*_ ∩ *U*. In particular, $\dot {\alpha }_{\ell }(t)= Z_{\ell }(t,p)$. We can build some 2-chains inside *U* bounded by these 1-chains as follows:

$$v_{\ell}(t,s) = \alpha_{\ell}(s) + t(\gamma_{\ell}(s)-\alpha_{\ell}(s)), $$

from [0,1] × [0,*ε*_*ℓ*_] to *U*. Since *α*_*ℓ*_(*s*) and *γ*_*ℓ*_(*s*) are piecewise *C*^1^ in the *s* variable, the domain of this map can be partitioned into smaller rectangles on which *v*_*ℓ*_(*t*, *s*) are differentiable and therefore whose images are two cells. Then for each *ℓ* the images of *v*_*ℓ*_(*t*, *s*) become 2-chains which we denote as *C*_*ℓ*_. Note that *v*_1_(*t*,0) = *v*_2_(*t*,0) = *q* for all *t*. And also let the image of *v*_2_(*t*, *ε*_2_) be a curve *τ*. Since $q_{1} \geqslant q_{2}$, image of *v*_1_(*t*, *ε*_2_) is *β* ⋅ *τ*. It is also clear that *v*_*ℓ*_(0,*s*) = *α*_*ℓ*_(*s*) and *v*_*ℓ*_(1,*s*) = *γ*_*ℓ*_(*s*). Then we orient these curves and *C*_*i*_ such that modulo some 1-chains whose image is *q*: *∂C*_1_ = *γ*_1_ − *β* − *τ* − *α*_1_, *∂C*_2_ = *τ* − *γ*_2_ + *α*_2_.

**Fig. 2:**
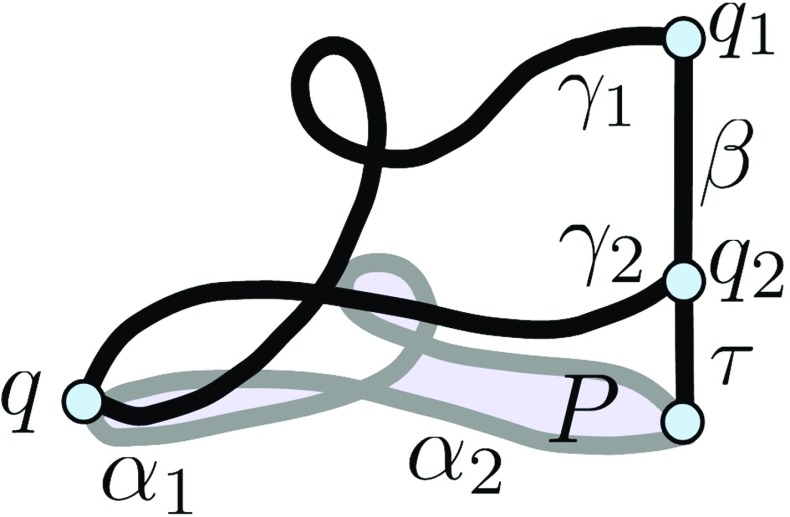
The closed 2-chain C

Let Γ be any 2-chain in *U* bounded by concatenating *γ*_1_, *γ*_2_ and *β* (whose composition is a 1-cycle in a contractible space so it always bounds a chain) in the right orientation so that *∂*Γ = *β* − *γ*_1_ + *γ*_2_. Finally, also orient *P* so that *∂P* = *α*_1_ − *α*_2_. Then, Γ,*C*_1_, *C*_2_ and *P* form a closed 2-chain *CC* (see Fig. 2). Using Stokes’ property and the fact that *∂CC* = *∅*, we get

$${\int}_{{\Gamma}}d\eta = -\left( {\int}_{P}d\eta + {\int}_{C_{1} \cup C_{2}}d\eta \right). $$

Moreover, since Γ is bounded by *γ*_1_, *γ*_2_ and *β* and $\eta _{\gamma _{i}(t)}(\dot {\gamma }_{i}(t))= 0$, we get again by Stokes’ property

2.8 $$ {\int}_{\beta}\eta={\int}_{{\Gamma}}d\eta = -\left( {\int}_{P}d\eta + {\int}_{C_{1} \cup C_{2}}d\eta \right).  $$

Defining $c= -{\int }_{C_{1} \cup C_{2}}d\eta $, we have

#### **Lemma 2.5**

*For* *β*, *c* *as defined above*

2.9 $$ |c| \leqslant C(n)\omega_{2\varepsilon}\varepsilon^{2}|d\eta|_{\infty},  $$

2.10 $$ |q_{2}-q_{1}||\eta(\partial_{y})|_{\inf} \leqslant |{\int}_{\beta} \eta | \leqslant |\eta(\partial_{y})|_{\infty}|q_{2}-q_{1}|.  $$

#### *Proof*

For the first inequality with $\dot {\alpha }(s)$ and $\dot {\gamma }(s)$ a.e. defined, we can write

$$\begin{array}{@{}rcl@{}} |{\int}_{C_{i}}d\eta | &\leqslant& {{\int}_{0}^{1}} dt {{\int}_{0}^{\varepsilon_{i}}} ds \ |d\eta|_{\infty} \underset{s \leqslant \varepsilon_{i}}{\sup} |\alpha_{i}(s) - \gamma_{i}(s)|((1-t)|\dot{\alpha}_{i}(s) | + t|\dot{\gamma}_{i}(s)|),\\ &\leqslant& 4\varepsilon_{i} |d\eta|_{\infty} \underset{s \leq \varepsilon_{i}}{\sup} |\alpha_{i}(s)-\gamma_{i}(s)|. \end{array} $$

Since $\dot {\alpha }_{i}(0)=\dot {\gamma }_{i}(0)$, $|\dot {\alpha }_{i}(t) - \dot {\gamma }_{i}(t)| \leqslant \tilde {C}\omega _{2\varepsilon _{i}}$ one has that $|\alpha _{i}(s)-\gamma _{i}(s)| \leqslant C(n)\varepsilon _{i} \omega _{2\varepsilon _{i}}$. So $|{\int }_{C_{i}}d\eta | \leqslant C(n)|d\eta |_{\infty } \omega _{2\varepsilon _{i}} {\varepsilon ^{2}_{i}} $ which implies $|c| =|{\int }_{C_{1} \cup C_{2}}d\eta | \leqslant C(n) \omega _{2\varepsilon } \varepsilon ^{2} |d\eta |_{\infty }$. The second part is an easy consequence of the fact that *η*_*p*_(*∂*_*y*_)≠ 0 for all *p* ∈ *U* and that $\dot {\beta }(t) \in \text {span} \langle \partial _{y} \rangle $.

Now using Eqs. 2.8, 2.9 and 2.10, we get that

$$\frac{1}{|\eta(\partial_{y})|_{\infty}}\left( {\int}_{P} d\eta - c\right) \leqslant |q_{2} - q_{1}| \leqslant \frac{1}{|\eta(\partial_{y})|_{\inf}}\left( {\int}_{P} d\eta + c\right), $$

where

$$|c|=|{\int}_{C_{1} \cup C_{2}}d\eta|\leqslant C(n)\omega_{2\varepsilon}\varepsilon^{2}|d\eta|_{\infty}. $$

The claim about the sign is also an immediate consequence of Eqs. 2.8 and 2.9. □

In the case *P* satisfies some properties, we can get a more precise estimate

#### **Corollary 2.6**

*Assume that* *P*, *γ*_1_, *γ*_2_, *q* *are as in* Proposition 2.4, *P* ⊂ *B*(*q*, *G*(*n*)*𝜖*_0_), *area of* P *is less than* *D*(*n*)*ε*^2^ *and* *ε* ≤ *F*(*n*)*𝜖*_0_ *for some* *D*(*n*),*G*(*n*),*F*(*n*) > 0. *Then for* *𝜖*_0_ *small enough, there exists a constant* *C*(*n*) *such that*

2.11 $$ |\gamma_{1}(\varepsilon_{1})-\gamma_{2}(\varepsilon_{2})| \leqslant C(n)\varepsilon^{2} \frac{|d\eta|_{{\Delta}}|_{\infty}}{|\eta(\partial_{y})|_{inf}}.  $$

*Moreover, if* *P* *is everywhere tangent to* *X*_*i*_(*q*), *X*_*j*_(*q*) *(q fixed)*,

2.12 $$ \frac{\varepsilon^{2}}{C(n)} \frac{|d\eta|_{{\Delta}}|_{inf}}{|\eta(\partial_{y})|_{\infty}} \leqslant |\gamma_{1}(\varepsilon_{1})-\gamma_{2}(\varepsilon_{2})|,  $$

*and*

2.13 $$ \quad sign(\gamma_{2}(\varepsilon_{2})-\gamma_{1}(\varepsilon_{1})) = sign\left( - d\eta_{q}(X_{i}(q),X_{j}(q)))\right).  $$

#### Proof

Since *P* is everywhere tangent to Δ_*q*_, $|{\int }_{P} d\eta | \leqslant a(P)\sup _{p \in P}|d\eta _{p}|_{{\Delta }_{q}}|$. Since Δ_*q*_ has modulus of continuity *ω*, we have that $|d\eta _{p}|_{{\Delta }_{q}}| \leqslant |d\eta _{p}|_{{\Delta }_{p}}| + E(n)\omega _{|p-q|}|d\eta |_{\infty }$ for some *E*(*n*) > 0. But |*p* − *q*|≤ *G*(*n*)*𝜖*_0_. Choose *𝜖*_0_ small enough so that $E(n)|d\eta |_{\infty }\omega _{G(n)\epsilon _{0}} \leqslant \frac {1}{4}|d\eta |_{{\Delta }}|_{\infty }$. Then, $|d\eta _{p}|_{{\Delta }_{q}}| \leqslant \frac {5}{4}|d\eta _{p}|_{{\Delta }_{p}}|$ and $|{\int }_{P} d\eta | \leqslant \frac {5}{4}D(n)\varepsilon ^{2}|d\eta _{{\Delta }}|_{\infty }$. Plugging this into Eq. 2.6 of previous proposition, we get

$$|\gamma_{1}(\varepsilon_{1})-\gamma_{2}(\varepsilon_{2})| \leqslant \frac{1}{|\eta(\partial_{y})|_{\infty}}\left( \frac{5}{4}D(n)\varepsilon^{2} |d\eta|_{{\Delta}}|_{\infty} + |c|\right). $$

Choose *𝜖*_0_ small enough so that $|c| \leqslant C(n) \varepsilon ^{2}\omega _{2F(n)\epsilon _{0}}|d\eta |_{\infty } \leqslant |d\eta |_{{\Delta }}|_{\infty }\varepsilon ^{2}$. Then, we get the first inequality. The second claim follows the same logic. Except now that *P* is everywhere tangent to *X*_*i*_(*q*),*X*_*j*_(*q*) for *q* is fixed. So in this case, we use the fact that (letting *p* ∈ *P*)

$$d\eta_{p}(X_{i}(q),X_{j}(q)) \geqslant d\eta(X_{i},X_{j})(p) - E(n)|d\eta|_{\infty}\omega_{G(n)\epsilon_{0}}. $$

Choose *𝜖*_0_ small enough so that $E(n)|d\eta |_{\infty }\omega _{G(n)\epsilon _{0}} \leqslant \frac {1}{4}|d\eta (X_{i},X_{j})|_{\inf }$. By condition in Eq. 2.10, *dη*_*p*_(*X*_*i*_(*p*),*X*_*j*_(*p*))≠ 0 and never changes sign then *dη*_*p*_(*X*_*i*_(*q*),*X*_*j*_(*q*)) never changes sign. Then choosing a parametrization *P*(*s*_*i*_, *s*_*j*_) such that $\frac {\partial P}{\partial s_{k}} = X_{k}(q)$ for *k* = *i*, *j*, one gets

2.14 $$ \left|{\int}_{P}d\eta\right| \geq {\int}^{a_{1}}_{0} {\int}^{a_{2}}_{0} |d\eta_{P(s)}(X_{i}(q),X_{j}(q))|ds_{1}ds_{2} \geq \frac{3}{4}\varepsilon^{2} D(n){|d\eta(X_{i},X_{j})|_{\inf}}.  $$

By condition in Eq. 2.3, $|d\eta (X_{i},X_{j})|_{\inf } \geqslant B(n)|d\eta _{{\Delta }}|_{\inf }|$, plug this in the equation above. Choose *𝜖*_0_ small enough so that $|c| \leqslant \frac {1}{4}B(n)D(n)|d\eta |_{{\Delta }}|_{\inf }\varepsilon ^{2}$. Plugging in this estimate for *c* and Eq. 2.14 into Eq. 2.6 of Proposition 2.4, we get the claim about the lower bound. For the claim regarding the sign, note that *η*(*∂*_*y*_) always has constant sign which wlog we can assume positive. So sign$(\gamma _{2}(\varepsilon _{2})-\gamma _{1}(\varepsilon _{1})) = \text {sign}({\int }_{\beta }\eta ) = \text {sign}\left (-{\int }_{P} d\eta + c\right )$. But by the above estimates on $|c| < |{\int }_{P}d\eta |$ so sign$\left (-{\int }_{P} d\eta + c\right ) = \text {sign}\left (-{\int }_{P} d\eta \right )=\text {sign}(-d\eta _{p}(X_{q},X_{q}))=\text {sign}(-d\eta _{q}(X_{i}(q),X_{j}(q))$ and we are done. □

### Proof of Theorem 1

The part of the Theorem 1 about smooth adapted coordinates is divided into two separate parts. First given *𝜖*, we will construct a certain *n*-dimensional manifold $\mathcal {W}_{\epsilon }$ using the adapted basis *X*_*i*_, which is admissible and transverse to *∂*_*y*_ direction. This is carried out in Section 2.3.1. Then. we will describe how the sub-Riemannian ball spreads around these manifolds in Section 2.3.2.

#### Part I: Construction of $\mathcal {W}_{\epsilon }$ and Its Properties

Given any |*𝜖*|≤ 2*n𝜖*_0_, due to condition in Eq. 2.4, we can define the *C*^1^ function *T*_*𝜖*_ : (−*𝜖*, *𝜖*)^*n*^ → *V*

$$T_{\epsilon}: (t_{1},\dots,t_{n}) \rightarrow e^{t_{n}X_{n}}\circ {\dots} \circ e^{t_{1}X_{1}}(0). $$

Notice also that it is 1-1 since *X*_*i*_ are uniquely integrable and since due to their form, an integral curve of *X*_*i*_ can intersect an integral curve of *X*_*j*_ for *i*≠*j* only once. Therefore, the image of *T*, which we denote as $\mathcal {W}_{\epsilon }$, is a *C*^1^ manifold. Moreover, every point on it is obviously accessible. Finally, it can be given as a graph over (*x*_1_,…,*x*_*n*_), in fact due to the form of the vector fields *T*_*𝜖*_(*x*_1_,…,*x*_*n*_) = (*x*_1_,…,*x*_*n*_, *a*(*x*_1_,…,*x*_*n*_)) for some *C*^1^ function *a*. In particular, note that if (*x*, *y*) = *T*_*𝜖*_(*t*_1_,…,*t*_*n*_), then |*x*| = |*t*|. Also $\mathcal {W}_{\epsilon } \subset U$. Two important properties that we will use often are given in the next lemma:

##### **Lemma 2.7**

*Let* $\mathcal {W}_{\epsilon }$ *be* *as above and* |*𝜖*|≤ 2*n𝜖*_0_. *Then,*

- *For any* *p* = (*x*, *y*) ∈ *U* *with* |*x*^*i*^|≤ *𝜖*, *there exists a unique* $q \in \mathcal {W}_{\epsilon }$ *with* *the same* *x coordinates as p*.
- *For any* $q=(x,y) \in \mathcal {W}_{\epsilon }$ *one* *has that* |*y*|≤|*x*|*C*(*n*)*ω*_2|*x*|_.

##### Proof

For the first item, taking *t*_*i*_ = *x*_*i*_ one has *T*_*𝜖*_(*t*_1_,…,*t*_*n*_) = (*t*_1_,…,*t*_*n*_, *a*(*t*_1_,…,*t*_*n*_)). For the second, letting q is the end point of composition of flows tangent to *X*_*i*_ which have modulus of continuity *ω*, total time of flow less than |*x*| and length less than 2|*x*|. Since the flow starts at the point 0, where *X*_*k*_(0) = *∂*_*k*_, its rise in y coordinates is bounded by |*x*|*C*(*n*)*ω*_2|*x*|_. □

#### Part II: Description of the Spread of *B*_Δ_(0,*𝜖*) Around $\mathcal {W}_{2\epsilon _{0}}$

Define for *p* = (*x*, *y*) with |*x*^*i*^|≤ *𝜖*, $d_{y}(p,\mathcal {W}_{\epsilon })$ to be the distance of *p* to the point *q* on $\mathcal {W}_{\epsilon }$ with the same *x* coordinate as *p*. For |*𝜖*|≤ *𝜖*_0_, we define the boxes

$$\mathcal{B}\mathcal{W}(K_{1}, \epsilon) = \{ p=(x,y) \in U, \ |x| \leqslant \epsilon \ \ s.t \ \ d_{y}(p,\mathcal{W}_{\epsilon}) \leqslant K_{1}\epsilon^{2}\}. $$

Note that these boxes are unions of $V= \{ |x| \leqslant \epsilon \} \cap \mathcal {W}_{\epsilon }$ and horizontal strips of length 2*K*_1_*𝜖*^2^ (which are inside *U* by conditions on *𝜖*_0_). Next, Lemma finalizes the proof of existence of smooth adapted coordinates in Theorem 2.

##### **Lemma 2.8**

*There exists an* *𝜖*_0_ > 0 *and* *K*_1_, *K*_2_ > 0 *such that* *for all* *𝜖* ≤ *𝜖*_0_ *one* *has,*

$$D_{2}^{\omega}\left( 0,K_{1}, \frac{\epsilon}{4}\right) \subset \mathcal{B}\mathcal{W}\left( K_{1}, \frac{\epsilon}{4}\right) \subset B_{{\Delta}}(0,\epsilon) \subset \mathcal{B}\mathcal{W}(K_{2}, 2n\epsilon) \subset H_{2}^{\omega}(0,K_{2},2n\epsilon). $$

##### *Proof of*$\mathcal {B}\mathcal {W}\left (K_{1}, \frac {\epsilon }{4}\right ) \subset B_{{\Delta }}(0,\epsilon )$

We start with the *𝜖*_0_ that has been fixed before Eq. 2.5. The proof is done if we show that (for some possibly smaller *𝜖*_0_) for all *𝜖* ≤ *𝜖*_0_, one has $V=\{|x| \leqslant \frac {\epsilon }{4}\} \cap \mathcal {W}_{\frac {\epsilon }{4}} \subset B_{{\Delta }}\left (0,\frac {\epsilon }{2}\right )$ and that starting on any point on *V*_*𝜖*_, we can travel with admissible curves of length less than or equal to $\frac {\epsilon }{2}$ along the directions ± *∂*_*y*_ up to some uniform amount $K_{1}\frac {1}{16}\epsilon ^{2}$. Then, concatenating these two curves, we get an admissible curve of length less than or equal to *𝜖* and reach anywhere in $\mathcal {B}\mathcal {W}\left (K_{1}, \frac {\epsilon }{4}\right )$. Constructing the first curve is easy since we know that by Lemma 2.7 and construction of $\mathcal {W}_{\epsilon }$, if $q_{1}=(x,y) \in \mathcal {W}_{\frac {\epsilon }{4}}$ is such that $|x| \leqslant \frac {\epsilon }{4}$, it is reachable by an admissible curve *τ* : [0,*𝜖*_*τ*_] → *U* with $\epsilon _{\tau } \leqslant \frac {\epsilon }{4}$ and length less than $\frac {\epsilon }{2}$.

Now, we build the curves that allows us to travel horizontally from any $q_{1} \in \mathcal {W}_{\frac {\epsilon }{4}}$. Consider the curves defined on $[0,{\tilde {\epsilon }}]$ for $\tilde {\epsilon } \leqslant \epsilon _{0}$ (the indexing of points below are chosen so that they are in direct alignment with Proposition 2.4):

$$\kappa_{1}(s)=e^{sX_{i}}(q_{1}) \quad \quad \kappa_{2}(s)= e^{sX_{j}}(\kappa_{1}({\tilde{\epsilon}})) \quad \quad \kappa_{2}({\tilde{\epsilon}})= q, $$

$$\kappa_{3}(s)=e^{-sX_{i}}(q) \quad \quad \kappa_{4}(s)= e^{-sX_{j}}(\kappa_{3}({\tilde{\epsilon}})) \quad \quad \kappa_{4}({\tilde{\epsilon}})= q_{2}. $$

**Fig. 3:**
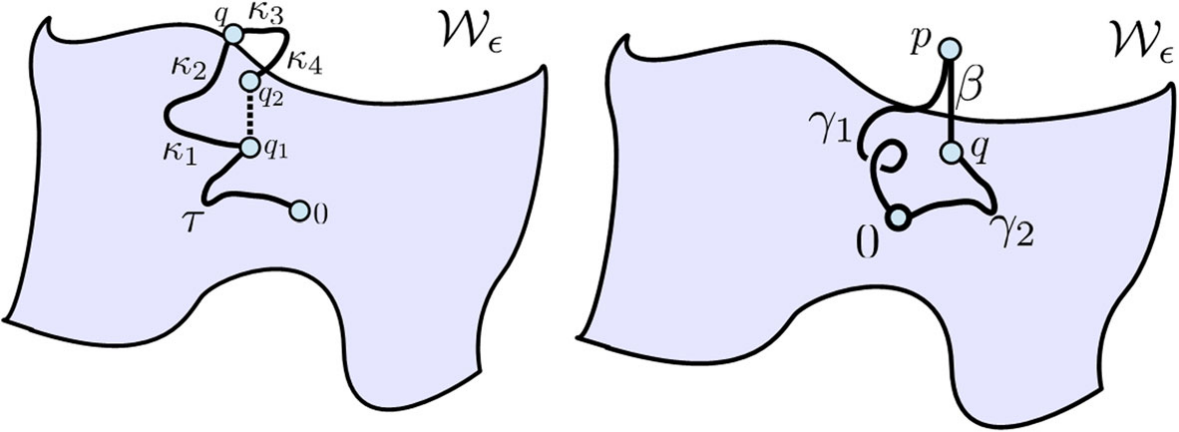
Admissible paths used in the proof of inclusions

We let $\gamma (s;{\tilde {\epsilon }},q_{1})$ be the parametrization for the curve obtained as the concatenation *κ*_4_ ∘ *κ*_3_ ∘ *κ*_2_ ∘ *κ*_1_, so that $\dot {\gamma }(s)=X_{\ell (s)}(\gamma (s;{\tilde {\epsilon }},q_{1}))$ for a.e. *s* with *ℓ*(*s*) = *i*, *j*. Also $q_{2}=\gamma (4{\tilde {\epsilon }}; {\tilde {\epsilon }}, q_{0})$ becomes the end point of this curve (see the left side of Fig. 3). This curve is defined on $[0,4{\tilde {\epsilon }}]$ to *U* and always lies on the *∂*_*y*_ axis passing above *q*_1_. Moreover, by Eq. 2.5, we have that $\gamma (s;{\tilde {\epsilon }},q_{0})$ is continuous with respect to $\tilde {\epsilon }$ since it is equal to $\gamma (s;\tilde {\epsilon },\tau ({\epsilon _{\tau }}))$ which is just a composition of *n* + 4 integral curves of some ± *X*_*ℓ*_ with integration times less than *𝜖*_0_. Denoting the image of this curve as $\gamma _{\tilde {\epsilon }}$, we have $\ell (\gamma _{\tilde {\epsilon }}) \leq 8\tilde {\epsilon }$. Therefore, to finish the proof, we need to show the following:

- There exists some *𝜖* ≤ *𝜖*_0_ such that setting $\tilde {\epsilon } = \frac {\epsilon }{16} |\gamma \left (\frac {\epsilon }{4};\frac {\epsilon }{16}\right ) - q_{1}| \geqslant K_{1} \frac {1}{16}\epsilon ^{2}$.
- By reverting either *X*_*i*_ or *X*_*j*_, we can go in the opposite direction along the *∂*_*y*_.

Note first that these are admissible curves which have length less than $8\frac {\epsilon }{16} = \frac {\epsilon }{2}$. Then, the last item allows us to travel in both directions (with respect to *∂*_*y*_) while the first item guarantees that we can travel more than |*p* − *q*_1_|. Then, by mean value theorem by reducing *𝜖*, we can reach any point on the *∂*_*y*_ axis passing through *q*_1_ whose distance to *q*_1_ is less than $K_{1}\frac {1}{16}\epsilon ^{2}$.

To apply Proposition 2.4, set $\gamma _{1} = {\kappa }_{1}^{-1} \circ {\kappa }_{2}^{-1}$, *γ*_2_ = *κ*_3_ ∘ *κ*_4_ (defined up to $\varepsilon _{i} = \frac {\epsilon }{16} \leqslant \frac {\epsilon _{0}}{16}$) which have lengths less than $\frac {\epsilon }{8}$, with *γ*_1_(0) = *γ*_2_(0) = *q* and Π_*x*_(*γ*_1_(*ε*_1_)) = Π_*x*_(*q*_1_) = Π_*x*_(*γ*_2_(*ε*_2_)) = Π_*x*_(*q*_2_). Projection of *γ*_*i*_ along *∂*_*y*_ to Δ_*q*_ gives us *α*_*i*_ the boundary of a parallelogram *P* with sides’ lengths less than $\frac {\epsilon }{8}$. Since *α*_*i*_ contain *q* and |*q*|≤ *𝜖*, they are inside *B*(0,2*𝜖*) ⊂ *U* (by condition in Eq. 2.4).

Now, it remains to show that *P* satisfies *P* ⊂Δ_*q*_ ∩ *U* and the conditions in Corollary 2.6. Given any point *z* ∈ *P*, |*z*|≤|*z* − *q*| + |*q*_1_ − *q*| + |*q*_1_|. But $|q_{1}| \leqslant 2|x| \leqslant \frac {\epsilon }{2}$, $|q_{1} - q| \leqslant \frac {\epsilon }{8}$, and $|z-q| \leqslant \frac {\epsilon }{4}$. So |*z*|≤ *𝜖* which by Eq. 2.4 gives us that *P* ⊂ *U*. Then, we also have that |*z* − *q*|≤|*z*| + |*q*|≤ *𝜖* + *ℓ*(*τ*) + *ℓ*(*γ*_1_) ≤ 2*𝜖*. Therefore, *P* ⊂ *B*(*q*, *G*(*n*)*𝜖*). Also area of *P* is less than *D*(*n*)*𝜖*^2^. So since $q_{2} = \gamma (\frac {\epsilon }{4}; \frac {\epsilon }{16}, q_{1}) = \gamma _{2}(\epsilon _{2})$ and *γ*_1_(*𝜖*_1_) = *q*_1_, using Eq. 2.12 in Corollary 2.6, we get

$$|\gamma \left( \frac{\epsilon}{4}; \frac{\epsilon}{16}, q_{1}\right) - q_{1}| \geqslant \frac{\epsilon^{2}}{C(n)} \frac{|d\eta|_{{\Delta}}|_{\inf}}{|\eta(\partial_{y})|_{\inf}}. $$

The claim about opposite directions is an automatic consequence of Eq. 2.13 of Corollary 2.6. □

##### *Proof of*$B_{{\Delta }}(0,\epsilon ) \subset \mathcal {B}\mathcal {W}(K_{2}, 2n\epsilon )$

To prove this inclusion, we need to prove that if *p* = (*x*, *y*) ∈ *U* such that *d*_Δ_(*p*,0) ≤ *𝜖*, then |*x*|≤ 2*n𝜖* and $d(p,\mathcal {W}_{2n\epsilon }) \leqslant K_{2}4n^{2}\epsilon ^{2}$ for some *K*_2_.

The condition *d*_Δ_(*p*,0) ≤ *𝜖* means that there exists an admissible curve *γ*_1_ : [0,2*𝜖*] → 0 that connects 0 to p such that *ℓ*(*γ*_1_) = 2*𝜖*. Since for all i, |*x*^*i*^|≤ *ℓ*(*γ*_1_) = 2*𝜖* we get |*x*|≤ 2*n𝜖*. So it remains to estimate $d(p,\mathcal {W}_{2n\epsilon })$.

The rest of the proof of this inclusion follows the method given in [12] by Gromov. The only essential difference is that the term |*dη*|_*∞*_ appearing there is replaced by |*dη*|_Δ_|_*∞*_ which will be thanks to Proposition 2.4. We first state a lemma (a more general version can be found in [11], Sublemma 3.4.B, see also Corollary 2.3 in [23] and a optimal version in [5] inside proof of Theorem 5.4.10):

##### **Lemma 2.9**

*Let c be a piecewise C*^1^, *closed curve in* $U \subset \mathbb {R}^{k}$ *with* $k \geqslant 2$ *and* *B*(*c*, *ℓ*(*c*)) ⊂ *U*. *Then, there exists a 2-chain cc such that* *∂*(*cc*) = *c*, *a*(*cc*) ≤ *C*(*n*)*ℓ*(*c*)^2^ *and* *cc* ⊂ *B*(*c*, *ℓ*(*c*)) *where* *a*(⋅) *denotes Euclidean area*.

Now since |*x*|≤ 2*n𝜖*, there exists a point q on $\mathcal {W}_{2n\epsilon }$ which has the same x coordinates as p. This means $d(p,\mathcal {W}_{2n\epsilon })=|q-p|$ and so we need to show that |*q* − *p*|≤ *K*_2_4*n*^2^*𝜖*^2^ for some *K*_2_. We can connect q to 0 by along $\mathcal {W}_{2n\epsilon }$ with an admissible path *γ*_2_ and apply Proposition 2.4 to the curves *γ*_1_, *γ*_2_ again (see right side of Fig. 3). We have *γ*_1_(0) = *γ*_2_(0) = 0 and *γ*_1_(*𝜖*_1_) = *p*, *γ*_2_(*𝜖*_2_) = *q* which have the same x coordinates. Then,

$$\ell(\gamma_{1}) \leqslant 2\epsilon \leqslant 2\epsilon_{0}, \quad \quad \ell(\gamma_{2}) \leqslant 2|x| \leqslant 4n\epsilon \leqslant 4n\epsilon_{0}, $$

$$\varepsilon_{1} = 2\epsilon , \quad \quad \varepsilon_{2} = |x| \leqslant 2n\epsilon, \quad \quad \varepsilon \leqslant 2n\epsilon \leqslant 2n\epsilon_{0} $$

The projection of *γ*_1_ and *γ*_2_ along *∂*_*y*_ to Δ_0_ forms a closed piecewise *C*^1^ curve $\alpha ={\alpha }_{1}^{-1} \circ \alpha _{2}$ which has length less than 2*𝜖* + 4*n𝜖* ≤ 6*n𝜖*. Since they also contain 0, these curves are in *B*(0,6*n𝜖*) which is in *U* by condition in Eq. 2.4. Now, we will build the surface *P* that satisfies the conditions in Proposition 2.4 and Corollary 2.6. By Eq. 2.4*B*(*α*, *ℓ*(*α*)) ⊂ *B*(0,18*n𝜖*) ⊂ *U*. So by Lemma 2.9, *α* bounds a 2-cycle *P* ⊂ *B*(0,18*n𝜖*) ⊂ *U* ∩Δ_0_ such that |*P*|≤ *C*(*n*)*𝜖*^2^. Since *γ*_1_(*𝜖*_1_) = *p* and *γ*_2_(*𝜖*_2_) = *q* we have by Eq. 2.11 of Corollary 2.6,

$$|p-q| \leqslant C(n)\epsilon^{2}\frac{{|d\eta|_{{\Delta}}|_{\infty}}}{|\eta(\partial_{y})|_{\inf}}, $$

which concludes the inclusion. □

#### Rest of the Proof of Proposition 2.8

Now, it is easy to prove the rest using Lemmas 2.7 and 2.8. Note again that for $q=(x,y) \in \mathcal {W}_{\epsilon }$, we have that $|y| \leqslant |x|\tilde {C}\omega _{2|x|}$.

First, we prove that $\mathcal {B}\mathcal {W}(K_{2}, 2n\epsilon ) \subset H_{2}^{\omega }(0,K_{2},2n\epsilon )$. If $(x,y) \in \mathcal {B}\mathcal {W}(K_{2}, 2n\epsilon )$, then |*x*|≤ 2*n𝜖* and $d(p,\mathcal {W}_{2n\epsilon }) \leqslant 4n^{2}\epsilon ^{2}$. This means there exists $q=(x,z) \in \mathcal {W}_{2n\epsilon }$ such that |*z* − *y*|≤ 4*K*_2_*n*^2^*𝜖*^2^. But we know that $|z| \leqslant |x|\tilde {C}\omega _{2|x|}$. So $|y| \leqslant |z| + |z-y| \leqslant 4n^{2}K_{2}\epsilon ^{2} + |x|\tilde {C}\omega _{2|x|}$ which means that $(x,y) \in H_{2}^{\omega }(0,K_{2},2n\epsilon )$.

Then, we prove ${D}_{2}^{\omega }\left (0,K_{1}, \frac {\epsilon }{4}\right ) \subset \mathcal {B}\mathcal {W}_{\epsilon _{0}}\left (K_{1}, \frac {\epsilon }{4}\right )$. If $p=(x,y) \in {D}_{2}^{\omega }\left (0,K_{1}, \frac {\epsilon }{4}\right )$, then $|x| + \sqrt {\frac {1}{K_{1}}(|x|\tilde {C}\omega _{2|x|} + |y|)} \leqslant \frac {\epsilon }{4}$. So we have $|x| \leqslant \frac {\epsilon }{4}$ and $(|x|\tilde {C}\omega _{2|x|} + |y|) \leqslant K_{1}\frac {\epsilon ^{2}}{16}$. Let $q=(x,z) \in \mathcal {W}_{\frac {\epsilon }{4}}$ with $|z| \leqslant |x|\tilde {C}\omega _{2|x|}$. Therefore, $|y| + |z| \leqslant K_{1}\frac {\epsilon ^{2}}{16}$. So $d\left (p,\mathcal {W}_{\frac {\epsilon }{4}}\right )= |p-q|=|y-z| \leqslant |y| + |z| \leqslant K_{1}\frac {\epsilon ^{2}}{16}$. This implies that $(x,y) \in \mathcal {B}\mathcal {W}\left (K_{1}, \frac {\epsilon }{4}\right )$.

#### *C*^1^ Adapted Coordinates

Finally, we prove the statement about the existence of *C*^1^ adapted coordinates. Note that for each *𝜖*, $\mathcal {W}_{\epsilon }$ are *C*^1^ surfaces given as the image of the map *T*_*𝜖*_(*t*_1_,…,*t*_*n*_) with |*t*_*i*_|≤ *𝜖* and for *𝜖*_1_ < *𝜖*_2_, $\mathcal {W}_{\epsilon _{1}} \subset \mathcal {W}_{\epsilon _{2}}$. Define the transformation *ϕ* : *V* → *U* on some appropriately sized domain *V* ⊂ *U* such that

2.15 $$ \phi(x,y) = (x,y-T_{2n\epsilon_{0}}(x)).  $$

Then, it is clear that this map is a *C*^1^ diffeomorphism onto its image and takes each $\mathcal {W}_{\epsilon } \cap V$ (for *𝜖* ≤ 2*n𝜖*_0_) to the *x* plane. It also maps *X*_*ℓ*_(0) to *∂*_*ℓ*_. This is again an adapted coordinate system. In particular in this adapted coordinate system $(x,y) \in \mathcal {B}\mathcal {W}_{2n\epsilon _{0}} (K, \epsilon )$ simply implies |*x*|≤ *𝜖* and |*y*|≤ *K𝜖*^2^. So we get $\mathcal {B}\mathcal {W}(K_{1}, \frac {\epsilon }{4}) = \mathcal {B}(0,K_{1}, \frac {\epsilon }{4})$ and $\mathcal {B}\mathcal {W}(K_{2}, 2n\epsilon )=\mathcal {B}_{2}(0,K_{2},2n\epsilon )$, which finishes the proof.

## Some Examples and Applications

In this section, we give some of the very basic properties of continuous exterior derivative ${{\Omega }^{1}_{d}}(M) \setminus {{\Omega }^{1}_{1}}(M)$. We believe that the theory of continuous exterior derivatives is much more richer, it for instance admits the Poincar/’e Lemma, chain complexes with *d*^2^ = 0 and usual homotopy theory, but it is out of the scope of this paper. These will be consider in a separate paper but the interested reader can have a look at the preprint [24].

### Some Properties of Continuous Exterior Derivative and Examples

To construct examples, we give an alternative characterization Lemma 3.1 which is proven in [14] (for the general case, see [24]).

#### **Lemma 3.1**

*Let* *η* *be a continuous differential 1-form defined on* *some* $V \subset \mathbb {R}^{m}$. *Then,* *η* *has a continuous exterior derivative* *dη* *on* V *if and only if for any open subsetU* ⊂ *V* *with compact closure* $\bar {U} \subset V$, *there exists a sequence of smooth differential 1 formsη*^*k*^ *defined on* U *such that* *η*^*k*^ *converges to* *η* *in* *C*^0^ *topology and* *dη*^*k*^ *converges to some differential (2)-form in* *C*^0^ *topology, which becomes the continuous exterior derivative ofη*.

Although this construction is local, one can show that the usual glueing techniques can be used to produce global examples. Since we are only interested in local examples here, interested read should refer to [24]. A class of examples is given by

#### **Lemma 3.2**

*Let* $\eta = {\sum }_{i = 1}^{n} a_{i}(x^{1},\dots ,x^{n})dx^{i}$ *such* *that each* *a*_*i*_ *is continuous and has continuous partial derivatives with respect to allx*^*j*^ *for* *j*≠*i*. *Then,* *η* *has continuous exterior derivative.*

#### Proof

By assumptions on regularity of the functions, we can find *C*^1^ functions (by mollification) ${{a}_{i}^{k}}$ that converge in *C*^0^ topology to *a*_*i*_, such that $\frac {\partial {{a}_{i}^{k}}}{\partial x^{j}}$ converges to $\frac {\partial a_{i}}{\partial x^{j}}$ for *i*≠*j*. Then, define

$$\eta^{k} = \sum\limits_{i = 1}^{n} {{a}_{i}^{k}}(x^{1},\dots,x^{n})dx^{i}, \quad and \quad d\eta^{k} = \sum\limits_{i,j = 1, i\neq j}^{n} \frac{\partial {{a}_{i}^{k}}}{\partial x^{j}}dx^{j} \wedge dx^{i}. $$

By assumption *η*^*k*^ converges in *C*^0^ topology to *η* and *dη*^*k*^ converges to $d\eta = {\sum }_{i,j = 1, i\neq j}^{n} \frac {\partial a_{i}}{\partial x^{j}}dx^{j} \wedge dx^{i}$. Therefore, *dη* is the continuous exterior derivative of *η* by Lemma 3.1. □

Now, we create an example from ${{\Omega }_{d}^{1}}(M) \setminus {{\Omega }_{1}^{1}}(M)$ which are non-integrable (therefore examples where our ball-box theorem applies but the others do not)

#### Example 3.3

We first consider the class of examples given by Lemma 3.2. We restrict our-self to $U \subset \mathbb {R}^{3}$ and write *η* = *ady* + *bdx* + *cdz*. Then since

$$d\eta = a_{x}dx \wedge dy + a_{z} dz \wedge dy +b_{y} dy \wedge dx + c_{y} dy \wedge dz +b_{z} dz \wedge dx +c_{x} dx \wedge dz, $$

the non-integrability condition becomes

$$\eta \wedge d\eta(p) =(-(a_{x} + b_{y})c + (a_{z} + c_{y})b - (b_{z}-c_{x})a)(p) dx^{1} \wedge dx^{2} \wedge dy \neq 0. $$

To construct a specific example, let us take *a*(*x*, *y*, *z*) = 1. To create a non-integrable and non-differentiable example, we have to satisfy (*c*_*x*_ − *b*_*z*_ + *b*_*y*_*c* − *c*_*y*_*b*)(*p*) > 0 at some point where *η* is non-differentiable. Consider

$$b(x,y,z) = \sin(y)e^{|x|^{\alpha}}z \quad \quad c(x,y,z) = \cos(y)e^{|z|^{\beta}}x, $$

for any 1 > *α*, *β* > 0, which gives

$$(c_{x}- b_{z}+ b_{y}c - c_{y}b)= \cos(y)e^{|z|^{\beta}}- \sin(y)e^{|x|^{\alpha}} + e^{|z|^{\beta} + |x|^{\alpha}}zx. $$

Then for instance, *η* (or any of its product with a differentiable function) is non-differentiable at *x* = 0,*z* = 0,*y* = 0 but *η* ∧ *dη*(0) = *dx* ∧ *dz* ∧ *dy*. Therefore, there exists a neighbourhood of 0 on which *η* is defined but non-differentiable and yet satisfies the sub-Riemannian properties mentioned in this paper. To create a non-Hölder example out of this, we can replace for instance $e^{|x|^{\frac {1}{2}}} $ with the function $f(x) = e^{\frac {1}{\log (|x|)}}$ (setting it to be 0 at 0) and we have an example that is non-Hölder at *x* = 0,*z* = 0,*y* = 0 and also non-integrable.

It is possible to given an example in which non of the components are differentiable in any variable but it requires to prove some analytic properties of continuous exterior derivative. We state the example without proof but the interested reader can see Example 2.17 and Lemma 2.15 in [24] for the proof of this example and further examples.

#### Example 3.4

Consider the following 1 −form

$$\eta = (z + 2)e^{|y|^{\frac{1}{2}}}dy + |x+z +y|^{\alpha} (dx + dz + dy) + xe^{y}dy + e^{y}dx. $$

All of the components of this one differential 1-form are not differentiable in any of the variables at the point 0, but one can show that it has continuous exterior derivative given by

$$d\eta = e^{|y|^{\frac{1}{2}}}dz \wedge dy, $$

and therefore,

$$\eta \wedge d\eta(0)= dx \wedge dz \wedge dy, $$

which satisfies the conditions of Theorem 1.

### Integrability of Bunched Partially Hyperbolic Systems

In this subsection, we give an application to dynamical systems.

Let *M* be a compact Riemannian manifold of dimension *n* + 1 and *f* : *M* → *M* is a *C*^3^ diffeomorphism. Assume moreover that there exists a continuous splitting $T_{x}M = {{E}_{x}^{s}} \oplus {{E}_{x}^{c}} \oplus {{E}_{x}^{u}}$, each of which is invariant under *Df*_*x*_. This splitting is called partially hyperbolic if there exists constants *K*, *λ*_*σ*_, *μ*_*σ*_ > 0 for *σ* = *s*, *c*, *u* such that *μ*_*s*_ ≤ 1, *μ*_*s*_ < *λ*_*c*_, *μ*_*c*_ < *λ*_*u*_, *λ*_*u*_ > 1 and for all *σ* = *s*, *c*, *u*, *x* ∈ *M* and *v*_*σ*_ ∈ *T*_*x*_*M* with |*v*_*σ*_| = 1 one has,

$$\frac{1}{K}{{\mu}_{s}^{i}} \leqslant |D{{f}_{x}^{i}} v_{\sigma}| \leqslant K{\lambda}_{\sigma}^{i} \quad \text{for all} \quad i>0 \ (i \in \mathbb{Z}^{+}). $$

This basically means that under iteration of *f*, the *Df*_*x*_ expands ${{E}_{x}^{u}}$ exponentially and contracts ${{E}_{x}^{s}}$ exponentially while the behaviour of ${{E}_{x}^{c}}$ is in between the two. Although these bundles might be just Hölder continuous (see [15]), a well-known property of such a system is that *E*^*u*^ and *E*^*s*^ are uniquely integrable into what are called as unstable and stable manifolds. Given *p* ∈ *U*, we denote the connected component of the stable and unstable manifold in *U* that contains *p* as ${\mathcal {W}}_{p}^{u}$ and ${\mathcal {W}}_{p}^{s}$ and call them local stable and unstable manifolds. In general *E*^*c*^, *E*^*cs*^ = *E*^*c*^ ⊕ *E*^*s*^ or *E*^*cu*^ = *E*^*c*^ ⊕ *E*^*u*^ maybe be non-integrable both in the case the bundles are continuous (see [16]) and or differentiable (see [7]). The integrability of these bundles play an important role in classification of the dynamics (see for instance [6, 13]). It is our aim to apply now Theorem 1 to get a novel criterion for integrability of these bundles under additional assumptions on geometry and dynamics.

A dynamical assumption that we will make is center bunching. A system is called center bunched if $\frac {{\lambda ^{2}_{c}}}{\mu _{u}}<1$. It means that the expansion in the unstable direction strongly dominates the expansion in center (as opposed to the definition of partially hyperbolic system where one only has $\frac {\lambda _{c}}{\mu _{u}}<1$). Conditions like center bunching appears quite commonly in studies of dynamical systems (see for instance [8] where center bunching plays an important role for ergodicity and [22] where a condition called pinching plays a role on regularity of the bundles). In Theorem 4.1 of [7], the authors prove that if *E*^*c*^ and *E*^*s*^ are *C*^1^ and center bunched, partially hyperbolic then *E*^*cs*^ is uniquely integrable. As far as we are aware, there is no general result on integrability of such continuous bundles that do not make any assumptions on the differentiable and topological properties of the manifold *M* (see for instance [5] where they assume *M* is a torus or [13] where they have assumptions on the fundamental group of the manifold). An integrability theorem for continuous bundles that only make assumptions on the constants above would indeed be quite useful. Since being a partially hyperbolic system and center bunched are preserved under *C*^1^ perturbations of *f*, they constitute an open set of examples (in *C*^1^ topology) inside partially hyperbolic systems.

Our aim is to make one small step towards an integrability condition for continuous bundles that relies only on the constants. The only place where differentiability is required in the proof of the theorem in [7] is where certain sub-Riemannian properties (more specifically the smaller box inclusion in the ball-box theorem) are required. Thus, once these properties are guaranteed then the proof easily carries through.

#### **Theorem 3**

*Assume* *f* : *M* → *M* *is a diffeomorphism of a compact manifold* *which admits a center bunched partially hyperbolic* *splitting* $T_{x}M = {{E}_{x}^{s}} \oplus {{E}_{x}^{c}} \oplus {{E}_{x}^{u}}$, *where* $dim({{E}_{x}^{u}})= 1$. *Assume moreover that* ${\mathcal {A}}_{0}^{1}(E^{cs})$ *admits* *a continuous exterior derivative. Then,* *E*^*cs*^ *is uniquely integrable with a* *C*^1^ *foliation.*

#### Proof

As in [7], one starts by assuming that there exists a point p where *η* ∧ *dη*(*p*) > 0 where *E*^*cs*^ = *ker*(*η*) and *dη* is the continuous exterior derivative. Then by Theorem 1, there exists a *C*^1^ adapted coordinate system and a neighbourhood U of p on which every point q on the local unstable manifold ${\mathcal {W}}_{p}^{u}$ of p can be connected to p by a length-parameterized admissible path *κ*(*t*) such that *κ*(0) = *p*, *κ*(*ℓ*(*κ*)) = *q* and *d*(*p*, *q*) ≥ *c*|*κ*|^2^. To show that the last condition can be achieved, we choose first a smooth adapted coordinate system at p for *E*^*sc*^ so that the unstable bundle is very close to the *∂*_*y*_ direction (since both are transverse to *E*^*sc*^ this is possible). By choosing it close enough, we can make sure that when we pass to the *C*^1^ adapted coordinates using the transformation given in Eq. 2.15, the unstable direction and *∂*_*y*_ direction are still close enough so that in a small enough neighbourhood U and for any $q=(x,y) \in {\mathcal {W}}_{p}^{u}$, |*x*^*i*^|≤ *δ*|*y*| where $\delta <\frac {1}{2n}$. Then to apply the ball-box theorem in this *C*^1^ adapted coordinate system, for *𝜖* small enough, we pick $q=(x,y) \in \mathcal {B}_{2}(0,K_{1},\epsilon )$ so that |*y*| = *K*_1_*𝜖*^2^. But the ball-box theorem tells us that there exists a length-parameterized admissible curve *κ* such that *ℓ*(*κ*) ≤ *𝜖*, *κ*(0) = *p* and *κ*(*ℓ*(*κ*)) = *q*. Since $|x^{i}| \leqslant \frac {1}{2n}|y|$, we have $d(q,p) \geqslant |y|-|x| \geqslant |y| - \frac {1}{2}|y| = \frac {1}{2}|y| = \frac {K_{1}}{2}{\epsilon ^{2}}$ (where in this coordinate system we remind that *p* = 0). Therefore, for some constant c, $d(q,p) \geqslant c|\kappa |^{2}$. Thus, the conditions 1 to 4 appearing in the proof of Theorem 4.1 of [7] are fully satisfied and the rest of the analysis only depends on the dynamics of f . So by the same contradiction obtained there, we get that *η* ∧ *dη* = 0 everywhere. Then, by the integrability theorem of Hartman in [14], this means that *E*^*cs*^ integrates to a unique *C*^1^ foliation. □

## Footnotes

1 The accessibility theorem for corank 1, step 2, completely non-integrable differentiable bundles was actually already formulated in 1909 by Carathéodory with the aim of studying adiabatic paths in thermodynamical systems [9]).

2 This notion dates back to some works of E. Cartan [10] and has been used in [14] and [19] to give continuous versions of Frobenius theorem.

3 We remind that a differentiable *n*-cell in *U* is a differentiable mapping from a convex *n*-polyhedron in $\mathbb {R}^{n}$ (with an orientation) to *U* and a *n*-chain is a formal sum of *n*-cells over integers. A cycle is a chain whose boundary is 0.
